# RNA G‐Quadruplexes in the Porcine Deltacoronavirus Genome: Structural Regulation and Therapeutic Targeting for Antiviral Strategies

**DOI:** 10.1155/tbed/5088147

**Published:** 2026-07-31

**Authors:** Jiajing Guo, Mengqi Yu, Yue Sun, Qiao Chen, Fei Liu, Yanke Shan

**Affiliations:** ^1^ College of Veterinary Medicine, Sanya Institute of Nanjing Agricultural University, Sanya 572000, Hainan, China; ^2^ Joint International Research Laboratory of Animal Health and Food Safety of Ministry of Education, Single Molecule Biochemistry and Biomedicine Laboratory (Sinmolab), Nanjing Agricultural University, Nanjing 210095, Jiangsu, China, njau.edu.cn

**Keywords:** antiviral activity, membrane proteins (M), nonstructural proteins 2 (Nsp2), porcine deltacoronavirus, RNA G-quadruplexes (RG4s)

## Abstract

Porcine deltacoronavirus (PDCoV) is an emerging enteric coronavirus that poses a considerable risk to swine production worldwide. G‐quadruplexes (G4s) are noncanonical nucleic acid secondary structures known to regulate critical steps of viral life cycles in multiple viruses; however, the existence and biological role of RNA G4s (RG4s) in PDCoV remain unclear. In this study, we identified putative G4‐forming sequences (PQSs) in the nonstructural proteins 2 (*Nsp2*) and *M* genes of the PDCoV genome, which are highly conserved across PDCoV strains, based on bioinformatic analysis. Fluorescence turn‐on assays and circular dichroism (CD) spectroscopy revealed that these PQSs fold into typical parallel RG4 structures in vitro, and their RG4 formation in living cells was further confirmed using G4‐specific probes. Notably, treatment with the G4 ligands TMPyP4 and pyridostatin (PDS) significantly reduced PDCoV *N* mRNA abundance and corresponding protein expression, concomitant with a marked decrease in viral titers in LLC‐PK1 cells. In addition, RG4 structures identified in the *Nsp2* and *M* mRNAs were shown to inhibit translation through a posttranscriptional mechanism. Moreover, G4 ligands, such as PDS and TMPyP4, targeted the RG4 structure in *Nsp2* mRNA and significantly inhibited Nsp2‐EGFP fusion protein expression without affecting the expression of the M‐EGFP fusion protein. In summary, our work supports the presence of sequence‐conserved functional RG4 structures in the PDCoV genome and provides new insights into the development of antiviral strategies targeting PDCoV RG4s.

## 1. Introduction

Porcine deltacoronavirus (PDCoV) is an emerging enteric coronavirus that causes acute watery diarrhea, vomiting, and dehydration in piglets, with mortality rates approaching 100% in neonatal cases [1–3]. First identified in 2012, PDCoV has since spread globally, inflicting substantial economic losses on the swine industry [4–6]. Alarmingly, recent reports of PDCoV infections in febrile children in Haiti suggest zoonotic potential, underscoring the critical importance of developing effective prevention and control measures [7]. However, the suboptimal effectiveness of existing vaccines or antiviral interventions against PDCoV underscores the importance of uncovering the molecular mechanisms that govern PDCoV replication and pathogenesis to identify novel therapeutic targets.

RNA G‐quadruplexes (RG4s) constitute noncanonical RNA secondary structures originating from guanine (G)‐rich sequences, characterized by two or more G‐quartet layers organized in a planar geometry stabilized by Hoogsteen hydrogen bonds [8, 9]. It contributes to viral RNA genome replication, transcription, and translation and represents a promising target for therapeutic intervention [10–12]. Although RNA viruses mutate rapidly, posing a persistent challenge to antiviral therapy [13, 14], RG4‐forming sequences have been identified in multiple viruses, including SARS‐CoV‐2 [15–17], PEDV [18], and PRRSV [19], and are often highly conserved across different strains of the same virus. Furthermore, viral RG4s are commonly positioned in genomic regulatory regions and participate in the regulation of essential viral activities [20–22]. For instance, several highly conserved and stable G‐quadruplex (G4) structures have been identified in SARS‐CoV‐2, which exert inhibitory effects on viral replication and translation. Among them, a stable RG4 structure was identified within the coding sequence (CDS) of the nucleocapsid phosphoprotein (N). This RG4 can be stabilized by ligands like PDP, causing significant suppression of N protein expression through translational inhibition [16]. Similarly, in PEDV, G4 ligands such as pyridostatin (PDS) and TMPyP4 target RG4 within the Nsp5 gene, suppressing Nsp5 protein expression and exhibiting antiviral activity during postinfection stages [18]. These studies further support that RG4 serves as critical regulatory elements in the life cycles of diverse RNA viruses, with coronaviruses as a representative example [17]. Nevertheless, despite extensive investigation of RG4s in various coronaviruses, their presence and biological functions remain unexplored in the emerging PDCoV. Given the high mutation rate of PDCoV, elucidating whether sequence‐conserved and functional RG4s also exist in its genome may reveal novel regulatory mechanisms and provide potential strategies for antiviral intervention.

In this study, we identified RG4‐forming sequences conserved across different PDCoV strains within the nonstructural proteins 2 (*Nsp2*) and *M* genes through bioinformatic screening and demonstrated that both sequences form parallel RG4 conformation in vitro and are capable of forming RG4 structures in living cells. Functional experiments revealed that the RG4 structures in the *Nsp2* and *M* genes significantly inhibit their translation. In addition, the G4 ligands PDS and TMPyP4 demonstrated anti‐PDCoV activity and inhibited Nsp2‐EGFP expression by targeting the RG4 in *Nsp2*, whereas the inhibitory effect on M‐EGFP expression was not significant. These findings provide a foundation for the development of potential anti‐PDCoV therapeutics targeting RG4 structures.

## 2. Materials and Methods

### 2.1. Oligonucleotides and Materials

All primer sequences and putative G4‐forming sequences (PQSs) utilized in this study were synthesized by GenScript (Nanjing, China), with their detailed information provided in Table S2. Thioflavin T (ThT), n‐methyl mesoporphyrin IX (NMM), TMPyP4, and PDS were purchased from MedChemExpress (MCE, China). BG4 antibody was obtained from Absolute Antibody (Ab0017424.1, UK). Mouse anti‐GFP antibody and GAPDH rabbit monoclonal antibody were obtained from ABclonal (China, AE012, A19056). Cell Counting Kit‐8 (C0038, CCK‐8) was acquired from Beyotime.

### 2.2. Cells and Viruses

HEK293T cells and LLC‐PK1 were kept in DMEM (Gibco, USA) and added with a concentration of 10% FBS (Proteintech, China). Both cell lines were subject to incubation in 5% CO_2_ at 37°C. LLC‐PK1 cells cultured in DMEM containing 5 μg/mL trypsin were used to propagate the PDCoV strain CZ2020 (GenBank accession number OK546242). After a 2 h infection period, the cells were rinsed and subsequently maintained in culture until pronounced cytopathic effects (CPEs) became evident. After three cycles of freezing and thawing of the infected cells, the supernatant obtained by centrifugation was collected as the viral stock.

### 2.3. Gel Mobility Shift Assay

FAM‐labeled RNA oligonucleotides were prepared at a concentration of 20 μM in a buffer containing 1 mM EDTA, 100 mM KCl, and 10 mM Tris‐HCl (pH 7.4). The samples were heated at 95°C for 5 min and then gradually cooled to room temperature. The folded RNA samples were then diluted to 25 nM before electrophoresis. For native PAGE analysis, the samples were electrophoresed on 10% native polyacrylamide gels prepared in 1× TBE buffer at 4°C in 1 × TBE running buffer containing 50 mM KCl under a constant voltage of 110 V for 1.5 h. In parallel, denaturing PAGE analysis was performed using 10% polyacrylamide gels containing 7 M urea, with 1× TBE running buffer containing urea. Fluorescence FAM‐labeled RNA bands were visualized using a Typhoon FLA 9500 imaging system (Cytiva).

### 2.4. Circular Dichroism (CD) Spectroscopy

The RNA oligonucleotides listed in Table S2 were dissolved at a 100 μM concentration in a buffer containing 1 mM EDTA, 1 M KCl, and 100 mM Tris‐HCl (pH 7.4). To promote RG4 formation under physiological ionic conditions, the RNAs were denatured at 95°C and subsequently allowed to cool slowly to room temperature. Secondary structures were characterized at room temperature using a Jasco J‐1500 spectropolarimeter. Spectra were recorded from 200–320 nm at a rate of 100 nm per minute. All measurements were baseline‐corrected against the buffer solution to ensure data reliability.

### 2.5. Fluorescence Turn‐On Assays

All assays were performed using a Tecan m200 Pro microplate reader. 1 μM RNA samples were initially denatured at 95°C for 5 min and subsequently allowed to cool gradually to 25°C. After incubation with either 5 μM NMM or 5 μM ThT, fluorescence emission was recorded with excitation wavelengths of 393 nm (NMM) and 425 nm (ThT).

### 2.6. Differential Scanning Calorimetry (DSC)

Thermal stability of Nsp2‐ and M‐PQSs was evaluated by DSC 9 (PerkinElmer). WT and mutant (Mut) RNA samples were prepared at 150 μM in KCl‐containing buffer, heated at 95°C, and gradually cooled to room temperature for annealing. For ligand‐binding experiments, TMPyP4 or PDS was added prior to thermal analysis. DSC measurements were performed using a heating rate of 1°C min^−1^. Melting temperatures (Tm) were determined from the maxima of the thermograms and used to assess the effects of sequence mutation and ligand binding on RG4 stability.

### 2.7. Fluorescence Polarization (FP) Assay

The binding affinities of TMPyP4 and PDS toward Nsp2‐ and M‐PQSs were determined by FP assays. FAM‐labeled WT and mutant RNA oligonucleotides were folded in buffer containing either 100 mM KCl or 100 mM LiCl, as described above. The final concentration of RNA was fixed at 10 nM, while TMPyP4 or PDS was added at concentrations ranging from 0–200 μM. After incubation at room temperature for 30 min, FP measurements were performed using a BioTek Synergy H1 microplate reader equipped with FP optics. FAM fluorescence was detected using the FITC/FAM channel, with excitation and emission wavelengths set at 485 and 528 nm, respectively. Parallel and perpendicular fluorescence intensities were recorded for FP calculation. Binding curves were fitted using a one‐site–specific binding model, and binding dissociation constants (*K*_*D*_) were calculated using GraphPad Prism 8.0.

### 2.8. Immunofluorescence Assays

The cells were seeded onto confocal dishes (NEST) precoated with a confluent monolayer. Following three washes with PBS, 2 μg of FAM‐labeled RNAs was transfected into the cells using Lipofectamine 3000 reagent. Cells were fixed with 4% paraformaldehyde for 15 min at 24 h posttransfection. Following PBS washes, cells were treated with 0.1% Triton X‐100 for permeabilization and 3% BSA for blocking. Subsequently, cells were incubated with BG4 primary antibody for 1 h, washed, and then exposed to Cy5‐labeled secondary antibody for 1 h. The nuclei were stained with DAPI for 10 min, and images were acquired using a Nikon laser‐scanning confocal microscope.

### 2.9. CCK8

The cytotoxicity of TMPyP4 and PDS on LLC‐PK1 cells was evaluated using CCK‐8. Briefly, cells were seeded into 96‐well plates and cultured until reaching confluent monolayers. After aspiration of the medium, cells were washed and exposed to maintenance medium containing serial dilutions of PDS or TMPyP4 (0–100 µM), and six replicate wells were used per concentration and incubated for 24 h. Subsequently, the medium was exchanged with DMEM supplemented with 10% CCK‐8 solution. After 2 h of additional incubation, measurements of optical density (OD) at 450 nm for each well were performed with a Tecan M200 Pro reader. Cell viability was calculated using the following formula:

Cell Viability %=Abssample−AbsblankAbsnegative control− Absblank×100%.

### 2.10. Virus Infection and RG4 Ligand Treatment

For TMPyP4 or PDS treatment, DMEM supplemented with RG4 ligands (0, 5, 10, and 20 μM) was added to LLC‐PK1 cells grown in 12‐well plates for 2 h. The treated cells were then infected with PDCoV at an MOI of 0.1 for 1 h. Following this, unattached viruses were removed, and 1 mL of DMEM supplemented with the corresponding drugs and trypsin (5 μg/mL) was added. At the designated time points, cells were harvested for subsequent analysis by quantitative Western blot, real‐time PCR, or TCID_50_.

### 2.11. Western Blot Analysis

Total protein extraction was performed with RIPA lysis buffer (Beyotime, China) and quantified via the BCA assay (Beyotime). Denatured protein samples were resolved on 10% SDS‐PAGE and transferred onto nitrocellulose membranes (Cytiva, USA). After blocking with 5% BSA for 1 h at 25°C, membranes were incubated with primary antibodies overnight at 4°C, followed by HRP‐conjugated secondary antibody incubation for 1 h at 25°C. Protein signals were detected using an ECL Western Blotting Substrate (Vazyme, China) and captured with chemiluminescence imaging.

### 2.12. RNA Extraction and qPCR

Total RNA was isolated using VeZol Reagent (Vazyme, R411), and then first‐strand cDNA was synthesized with the HiScript II kit (Vazyme, R211‐01), according to the respective manufacturer’s protocols. qPCR was carried out using ChamQ SYBR qPCR Master Mix (Q321, Vazyme) with gene‐specific primers (listed in Table S2) on a real‐time PCR system. The expression of mRNA was normalized against the endogenous reference GAPDH, and relative levels were calculated employing the 2^-ΔΔCt^ method.

### 2.13. TCID_50_

For the measurement of viral titers, 96‐well plates with cell monolayers were treated with 100 μL of PDCoV suspension per well. Eight replicates were performed for each dilution. The plates were incubated for 3–5 days at 37°C with 5% CO_2_, and CPEs were subsequently assessed and recorded. The TCID_50_ was calculated using the Reed–Muench method based on the proportion of CPE‐positive wells across serial dilutions.

### 2.14. Plasmids and Transfection

The CDSs of the PDCoV *Nsp2* and *M* genes were PCR‐amplified and cloned into the pEGFP‐N1 vector. To disrupt RG4 formation, guanines (Gs) in the G‐tracts were replaced with adenines or thymines (A/T) to generate the RG4 Mut construct, ensuring the elimination of synonymous substitutions that might form RG4. Cells were seeded in 12‐well plates and transfected using Lipofectamine 3000 (Invitrogen, USA) with 1 μg of plasmid. At 24 h posttransfection, cells were harvested for RNA extraction (TRIzol reagent) or protein lysis (RIPA buffer) for subsequent qPCR and Western blot analyses. Cells were incubated with the G4 ligands TMPyP4 or PDS at final concentrations of 0, 5, 10, and 20 μM and maintained for 24 h before harvesting.

### 2.15. Flow Cytometry Analysis

Flow cytometry was employed to quantify cells that had been transfected with the corresponding expression vector. At 24 h posttransfection, cells were trypsinized, harvested by washing, and centrifuged. Cell pellets were resuspended in 4% paraformaldehyde and fixed. After fixation, cells were washed to remove residual fixative. Flow cytometry was conducted on a BD FACSVerse instrument utilizing a 488 nm laser to excite EGFP. Data acquisition and analysis were conducted with BD FACSuite software, with a minimum of 10,000 events recorded per sample. Untransfected cells were used to establish background fluorescence thresholds.

### 2.16. Statistical Analysis

All statistical analyses were conducted by GraphPad Prism (version 8.0). Group differences were evaluated for statistical significance using one‐way analysis of variance (ANOVA). Data are presented as mean ± SEM. Asterisks in the panels flag the attained significance levels (ns = not significant;  ^∗^ *p*  < 0.05;  ^∗∗^ *p*  < 0.01;  ^∗∗∗^ *p*  < 0.001).

## 3. Results

### 3.1. Bioinformatic Analysis of Conserved RG4s in PDCoV Genome

**Figure 1:**
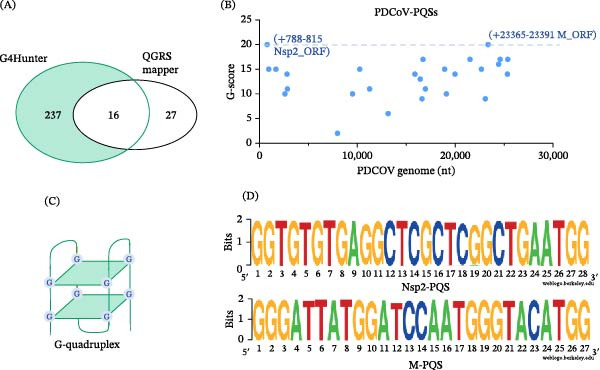
Bioinformatic analysis of potential RG4 sequences in PDCoV genome. (A) Venn diagram showing overlapping predictions of PQSs by QGRS mapper and G4Hunter. (B) Positional distribution and prediction scores of identified PQSs in the PDCoV genome. (C) Two G‐quadruplex layers are arranged in a stacked configuration, forming a noncanonical secondary structure known as a G‐tetrad. (D) Sequence conservation analysis of G‐rich regions capable of forming RG4 structures.

To map potential RNA RG4 structures across the PDCoV genome, genome‐wide predictions were conducted using two complementary algorithms, QGRS Mapper [23] and G4Hunter [24]. Cross‐comparison of predictions from both algorithms identified 16 putative PQSs consistently detected by both tools, distributed throughout the viral genome (Table S1). Among these candidates, two PQSs consistently ranked as the highest‐confidence motifs in both algorithms (G‐score ≥ 20), positioned within *Nsp2* (+788 to +815 relative to the ORF1a start codon) and the *M* gene (+23365 to +23391), respectively (Figure 1A–C).

Subsequent comparative analysis of 190 publicly available PDCoV genomes revealed that both high‐scoring PQSs are highly conserved across PDCoV isolates, as shown in the sequence logos (Figure 1D). Their high conservation, together with their potential to form RG4s, suggests that these PQSs may play essential regulatory roles during PDCoV replication.

### 3.2. Biophysical Characterization of RG4 Formation

**Figure 2:**
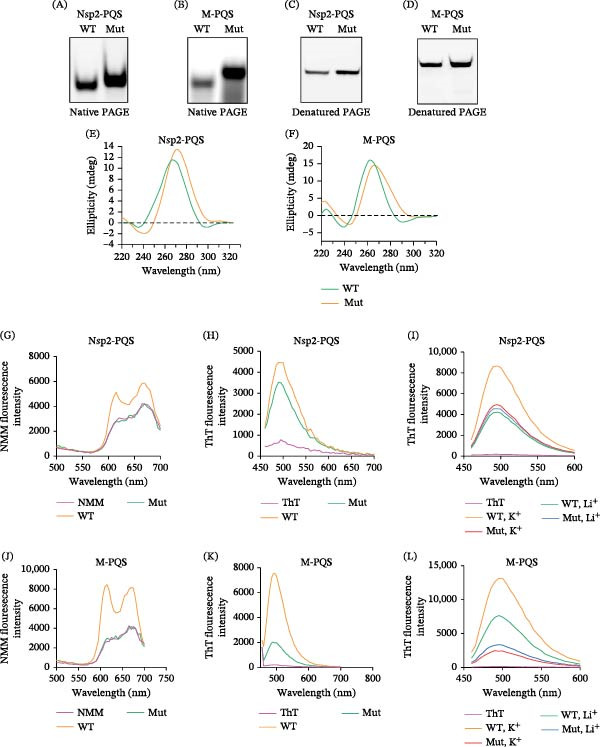
In vitro confirmation and characterization of PDCoV RG4 formation. (A) Native PAGE analysis of Nsp2‐PQS‐WT and Mut. (B) Native PAGE analysis of M‐PQS‐WT and Mut. (C) Denaturing PAGE analysis of Nsp2‐PQS‐WT and Mut. (D) Denaturing PAGE analysis of M‐PQS‐WT and Mut. (E, F) CD spectra of Nsp2‐PQS‐WT/Mut and M‐PQS‐WT/Mut in 100 mM KCl. (G) NMM fluorescence spectra of Nsp2‐PQS‐WT and Mut. (H) ThT fluorescence spectra of Nsp2‐PQS‐WT and Mut. (I) ThT fluorescence spectra of Nsp2‐PQS‐WT and Mut under KCl or LiCl conditions. (J) NMM fluorescence spectra of M‐PQS‐WT and Mut. (K) ThT fluorescence spectra of M‐PQS‐WT and Mut. (L) ThT fluorescence spectra of M‐PQS‐WT and Mut under KCl or LiCl conditions.

To investigate whether the predicted PQSs in *Nsp2* and *M* genes form RG4s, we analyzed wild‐type (WT) and G4‐disruptive Mut oligonucleotides by native PAGE. The oligonucleotides (Nsp2‐ and M‐PQS‐WT and Nsp2‐ and M‐PQS‐Mut) were subjected to folding in buffer with 100 mM KCl to induce RG4 formation. Under native conditions, WT oligonucleotides showed markedly altered electrophoretic mobility compared to their Muts, indicating conformational differences. In contrast, all sequences comigrated as single strands under denaturing conditions, consistent with identical primary sequence lengths (Figure 2A–D).

We next used CD spectroscopy to further probe their secondary structure. Both WT oligonucleotides showed the signature spectral profile of parallel RG4s, marked by a distinct positive peak at ~260 nm and a corresponding negative peak near 240 nm. By comparison, the Mut variants showed substantially altered CD profiles, confirming the disruption of RG4 folding (Figure 2E,F). Together, these results confirm that PQSs in *Nsp2* and *M* form RG4s in vitro and that intact G‐rich motifs are essential for proper RG4 folding.

To further validate RG4 formation and determine the ionic conditions favorable for RG4 folding, we employed the G4‐specific probes NMM [25] and ThT [26]. A significant fluorescence enhancement was observed for both WT sequences in KCl‐containing buffer, whereas the Mut controls (Nsp2‐ and M‐PQS‐Mut) exhibited minimal response (Figure 2G,H,J,K). Further probing into the ionic preference revealed that LiCl was insufficient to promote significant RG4 folding, as evidenced by a much lower fluorescence intensity in LiCl buffer versus KCl buffer (Figure 2I,L). These findings suggest that Nsp2‐ and M‐PQS‐WT form the classical RG4 conformation and that K^+^ provides the optimal ionic environment for RG4.

#### 3.2.1. Binding of G4 Ligands to Nsp2 and M RG4s

**Figure 3:**
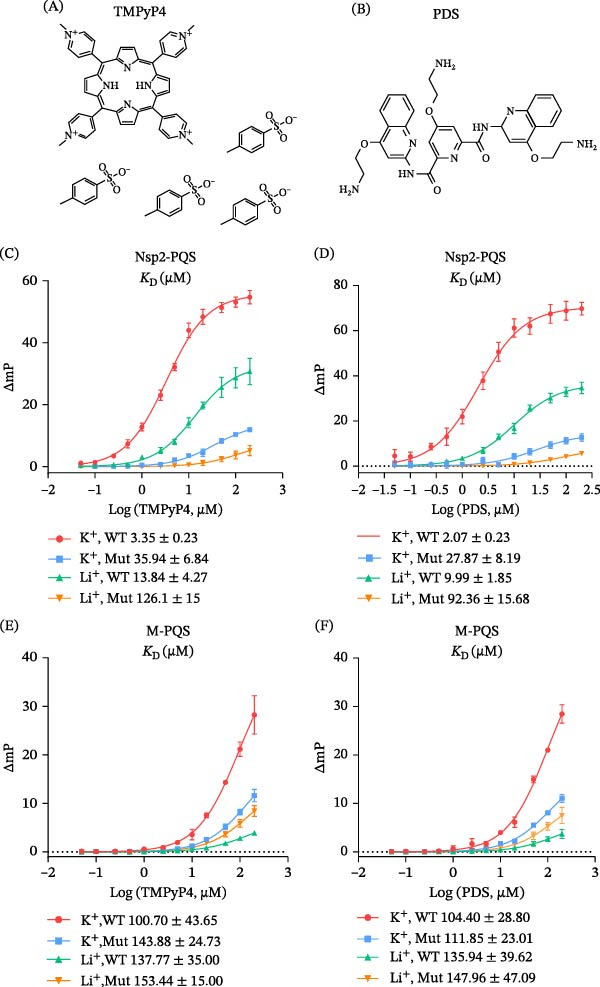
Binding of G4 ligands to Nsp2 and M RG4s. (A, B) Chemical structures of TMPyP4 and PDS. (C, D) Fluorescence polarization binding curves of TMPyP4 and PDS with Nsp2‐PQS‐WT/Mut under KCl or LiCl conditions. (E, F) Fluorescence polarization binding curves of TMPyP4 and PDS with M‐PQS‐WT/Mut under KCl or LiCl conditions. FAM‐labeled RNA oligonucleotides were used at a fixed concentration of 10 nM, and ligand concentrations ranged from 0 to 200 μM. Binding curves were fitted using a one‐site–specific binding model to calculate *K*_*D*_ values.

To evaluate the interactions between RG4s and G4 ligands, we measured the binding affinities of TMPyP4 and PDS toward Nsp2‐PQS and M‐PQS using FP assays (Figure 3).

For Nsp2‐PQS, both TMPyP4 and PDS exhibited strong binding to the WT sequence under KCl conditions, with *K*_*D*_ values of 3.35 μM ± 0.23 and 2.07 μM ± 0.23, respectively (Figure 3C,D). In contrast, ligand binding was markedly reduced in the Mut sequence and under LiCl conditions, indicating that the interaction depends on RG4 formation. Compared with Nsp2‐PQS, both ligands displayed substantially weaker binding to M‐PQS, with *K*_*D*_ values exceeding 100 μM under all tested conditions (Figure 3E,F). Moreover, neither the Mut sequence nor LiCl conditions enhanced ligand binding.

Taken together, TMPyP4 and PDS preferentially recognize Nsp2‐PQS WT RG4 structures formed under KCl conditions, whereas their binding affinities are markedly reduced for Mut sequences and under LiCl conditions, indicating that ligand binding is highly dependent on RG4 formation and a favorable K^+^ ionic environment.

### 3.3. Thermal Stabilization of RG4s by G4 Ligands

To examine the effects of G4 ligands on RG4 stability, thermal unfolding profiles of Nsp2‐ and M‐PQSs were analyzed by DSC (Figure S1 and Table 1).

**Table 1:** Melting temperatures (Tm) of Nsp2‐ and M‐RG4s determined by DSC.

Group	Nsp2 Tm (°C)	M Tm (°C)
WT	61.2	71.4
Mut	52.8	64.6
WT, TMPyP4	69.2	72.4
Mut, TMPyP4	53.8	66.2
WT, PDS	67.6	71.0
Mut, PDS	59.6	66.0

For Nsp2‐PQS, the WT sequence exhibited a Tm of 61.2°C, whereas disruption of the G‐rich motif reduced the Tm to 52.8°C, indicating that RG4 formation contributes substantially to thermal stability. The addition of TMPyP4 or PDS increased the Tm of Nsp2‐PQS‐WT to 69.2°C and 67.6°C, respectively, corresponding to increases of 8.0°C and 6.4°C relative to the untreated WT sequence. In contrast, neither ligand increased the thermal stability of the Mut sequence (Table 1).

In comparison, ligand treatment resulted in only minor changes in the Tm of M‐PQS. The Mut sequence showed a similar trend (Table 1). These results demonstrate that TMPyP4 and PDS selectively stabilize the RG4 formed by Nsp2‐PQS, whereas their effects on M‐PQS are limited.

### 3.4. Detection of RG4 Formation in Nsp2/M‐PQS Within Live Cells

**Figure 4:**
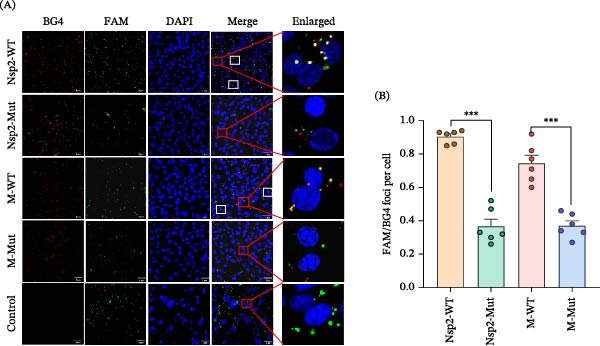
RG4 structures formed in the *Nsp2*/*M* genes of PDCoV in living cells. (A) Confocal immunofluorescence images showing colocalization of Nsp2‐ and M‐WT/Mut RNA (green) with BG4 antibody (red) in LLC‐PK1 cells. Nuclei were counterstained with DAPI (blue). White boxes indicate representative FAM‐RNA/BG4 colocalization foci in the merged images, shown as yellow puncta, and the selected regions are enlarged on the right. Scale bar: 5 μm. (B) Quantification of FAM‐RNA/BG4 colocalization foci.  ^∗∗∗^ *p* ≤ 0.001.

In vitro validation confirmed RG4 formation; we next examined whether these structures form intracellularly [27]. The FAM‐labeled WT Nsp2‐ and M‐PQS sequences exhibited clear RG4 formation, as evidenced by strong colocalization with BG4 (orange signal, Figure 4A). In contrast, the FAM‐labeled Nsp2‐and M‐PQS‐Mut displayed negligible colocalization with BG4 (Figure 4B). These results indicate that the WT PQS sequences in *Nsp2* and *M* can form RG4 structures in cells.

### 3.5. RG4 Ligands Inhibit PDCoV Infection In Vitro

**Figure 5:**
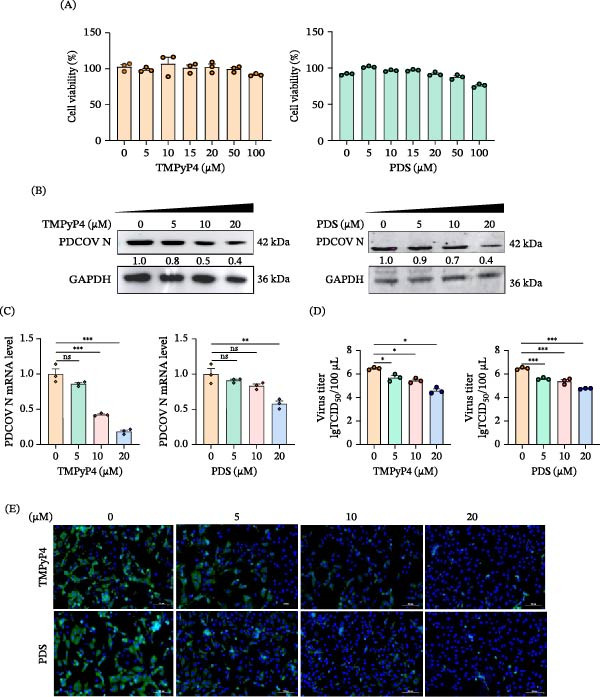
G4 ligands TMPyP4 and PDS suppress PDCoV replication. (A) Cytotoxicity of TMPyP4 and PDS in LLC‐PK1 cells were evaluated using a CCK‐8 assay. (B) LLC‐PK1 cells were incubated with varying concentrations of TMPyP4 or PDS prior to infection with PDCoV at an MOI of 0.1. After 12 h, total cell lysates were harvested, and PDCoV N protein levels were analyzed by WB. (C) Cell samples treated with TMPyP4 or PDS were collected for extraction, and PDCoV replication was quantified using qPCR. (D) The inhibitory effect of two ligands on PDCoV was determined by TCID_50_ assay. (E) Immunofluorescence was used to assess PDCoV proliferation after 24 h of treatment with specified doses of PDS or TMPyP4 (green: viral protein; blue: DAPI‐stained nuclei).

To examine whether G4 ligands could suppress PDCoV replication, two well‐characterized G4 ligands (TMPyP4 and PDS) [28, 29] were selected for analysis. The cytotoxicity of both compounds in LLC‐PK1 cells was first assessed, revealing minimal toxicity at concentrations ≤ 100 μM (Figure 5A) [18]. We next examined their effects on PDCoV replication. The dose‐dependent suppression of viral replication was confirmed at both the protein and transcript levels, with Western blotting revealing a significant reduction in N protein expression at 20 μM (Figure 5B) and qPCR showing a concomitant decrease in *N* mRNA abundance (Figure 5C). The dose‐dependent inhibitory effect was further quantified by determining viral titers using the TCID_50_ assay, which showed a significant reduction in infectious virus production upon treatment, aligning with the trends observed in protein and RNA analyses (Figure 5D). The immunofluorescence experiment confirmed these findings, showing that the fluorescence intensity of the viral N protein significantly decreased after ligand treatment, thereby confirming its effective inhibitory effect on viral replication (Figure 5E). Collectively, these results establish that TMPyP4 and PDS effectively suppress PDCoV proliferation in LLC‐PK1 cells, providing experimental evidence for their antiviral potential.

### 3.6. RG4 Structures Suppress Protein Translation at the Posttranscriptional Level in Cells

**Figure 6:**
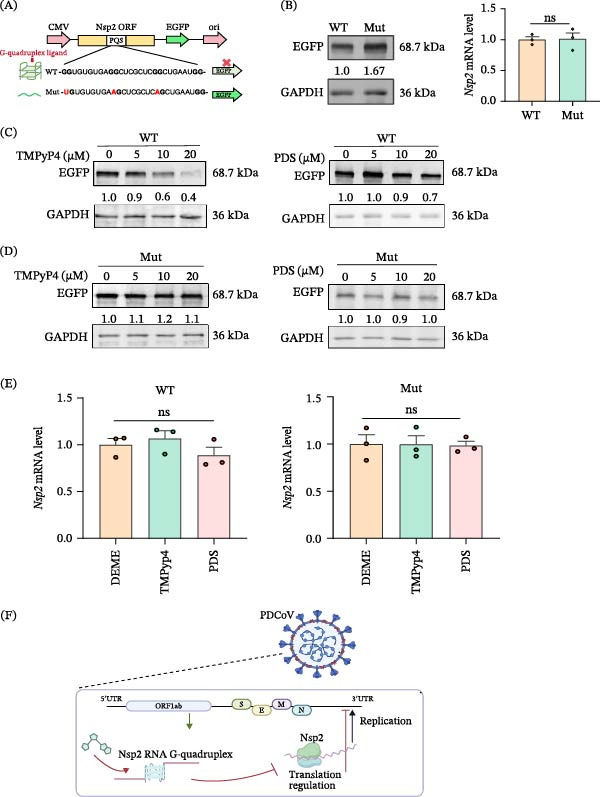
Posttranscriptional regulation of Nsp2 expression by RG4. (A) Schematic of *Nsp2* full‐length ORF or RG4 mutant constructs cloned into the MCS of pEGFP‐N1. Bold letters indicate RG4‐forming sequences; red letters denote mutations. (B) Nsp2‐EGFP protein levels (left) and mRNA (right) expression in cells following transfection with either pEGFP‐Nsp2‐WT or Mut plasmids. (C) The positions of the concentration and grayscale value numbers in the figure are as closely aligned with the band positions as possible. (D) Protein levels in cells treated with increasing concentrations of TMPyP4 (left) or PDS (right) for 24 h. (E) qPCR quantification of pEGFP‐Nsp2‐WT (left) or Mut (right) mRNA levels in cells treated with G4 ligands (20 μM) for 24 h. (F) Schematic model illustrating RG4 regulation during PDCoV infection.

To evaluate whether PDCoV RG4 formation regulates protein expression in cells, we constructed EGFP‐tagged WT and G4‐disruptive Mut reporter variants containing the *Nsp2* and *M* gene segments (Figure 6A and Supporting Figure S2A). Upon transfection of these reporter constructs into cells, disruption of the RG4 significantly enhanced Nsp2‐EGFP and M‐EGFP protein expression, whereas the corresponding mRNA levels remained unchanged (Figure 6B and Figure S2B). This discordance between increased protein abundance and unchanged mRNA levels highlights that the inhibitory effect of RG4s occurs primarily at the posttranscriptional level, most likely by reducing translation efficiency. Dose‐dependent experiments showed that both TMPyP4 and PDS inhibited Nsp2‐EGFP expression in a concentration‐dependent manner (Figure 6C), whereas their effects on M‐EGFP expression were significantly weaker (Figure S2C). Neither compound affected EGFP expression in cells transfected with the RG4‐Mut Nsp2 construct (Figure 6D), and the mRNA levels remained unchanged under all conditions (Figure 6E and Figure S2E). These results indicate that the inhibitory effect of G4 ligands on Nsp2‐EGFP expression occurs at the posttranscriptional level and depends specifically on an intact RG4 structure in *Nsp2*. In addition, no inhibitory effect of either ligand on M‐EGFP expression was observed (Figure S2D). Given that both G4 ligands exhibited minimal impact on M protein levels, we subsequently focused our flow cytometry analysis on Nsp2 for further investigation. Flow cytometry was employed to quantitatively analyze the cell populations, with assessment focusing on two key parameters: the percentage of EGFP‐positive cells and the mean fluorescence intensity (MFI). Experimental data demonstrated that in cells transfected with the pEGFP‐Nsp2‐WT reporter construct, treatment with each of the two G4 ligands resulted in a significant suppression of EGFP protein expression (Figure S3A). This was specifically characterized by a substantial reduction in MFI (Figure S3C) and a marked decrease in the proportion of EGFP‐positive cells (Figure S3B). In contrast, no significant changes were observed in cells transfected with the pEGFP‐Nsp2‐Mut construct. These findings indicate that the ligand‐induced suppression of Nsp2‐EGFP expression is strictly dependent on the structural integrity of RG4 (Figure S3A–C). Collectively, these results suggest that the G4 ligands inhibit the expression of Nsp2 by targeting the RG4 structure.

## 4. Discussion

Viral genomic RG4s have emerged as a novel class of antiviral targets, with their functions and therapeutic potential validated in coronaviruses such as SARS‐CoV‐2 [30–32] and PEDV [18]. However, the presence and functional significance of RG4s in PDCoV have not yet been investigated, despite PDCoV constituting a substantial risk to swine production. In this study, by integrating bioinformatic, biochemical, and biophysical approaches, we identified conserved parallel RG4 structures within the PDCoV *Nsp2* and *M* genes and confirmed their formation in living cells for the first time. Notably, stabilization of RG4 in *Nsp2* mRNA by G4 ligands inhibited Nsp2‐EGFP expression and exerted antiviral activity. Collectively, these findings indicate that RG4 structures may serve as emerging antiviral targets and that G4 ligands hold promise as an intervention against PDCoV infection.

Our study reveals that the RG4 structures within the *Nsp2* and *M* genes significantly suppress their translation, with this inhibitory effect depending on the integrity of the RG4 structure and occurring at the posttranscriptional level. This observation is consistent with RG4‐mediated translational regulation reported in various coronaviruses and eukaryotic mRNAs [33, 34] and may partially explain the antiviral effects observed in infected cells. In the specific context of PDCoV, our newly added FP (Figure 3) and DSC (Table 1) analyses further support a structure‐dependent model of translational repression, particularly showing that TMPyP4 and PDS directly bind to and stabilize the Nsp2 RG4 structure. Meanwhile, disruption of RG4 markedly increased protein expression without altering the corresponding mRNA abundance. Together, these findings suggest that folded RG4 may act as a local RNA structural barrier that impedes ribosome progression or translational elongation along the mRNA, thereby reducing translation efficiency. In future studies, direct transfection of in vitro transcribed WT and RG4‐Mut mRNAs would provide an additional strategy to further exclude transcription‐related effects and more directly validate the posttranscriptional role of RG4s in regulating translation.

However, in addition to this direct structural barrier model, the possible involvement of host RNA‐binding proteins in RG4‐mediated regulation should also be considered. Previous studies have highlighted the important roles of host factors in viral RG4 regulation [35–37]. For example, hnRNPH1 promotes viral replication by binding to and reducing the stability of viral RG4s [38]. Similarly, in SARS‐CoV and SARS‐CoV‐2, the conserved SUD domain within Nsp3 can bind both host‐ and virus‐derived RG4s and modulate the activity of viral replication/transcription complexes, while this interaction can be disrupted by G4‐stabilizing ligands [39]. These findings suggest that RG4s may serve as key regulatory hubs within the host–pathogen interaction network [40]. Therefore, future studies combining RNA pull‐down assays with proteomic analyses will be important to identify proteins interacting with PDCoV RG4s and to further determine whether these factors cooperate with or counteract RG4‐mediated translational repression and whether such effects are achieved by stabilizing or remodeling RG4 conformations.

Notably, among the two RG4‐containing reporter systems examined, Nsp2‐EGFP expression was significantly inhibited by both G4 ligands, whereas M‐EGFP showed weak responsiveness to ligand treatment. This differential response is consistent with the stronger binding affinity and thermal stabilization observed for Nsp2 RG4 compared with M RG4. Given that Nsp2 has been reported to promote immune evasion in multiple coronaviruses by interfering with signaling pathways or degrading immune‐related molecules [41–43] and specifically suppresses the interferon response in PDCoV by degrading STING [44], targeting the RG4 structure within *Nsp2* mRNA may reduce Nsp2 protein expression and thereby indirectly mitigate viral immune evasion. In future studies, the generation of polyclonal antibodies against PDCoV Nsp2 would be valuable for further examining the effects of RG4 formation and G4 ligand treatment on endogenous Nsp2 expression during viral infection, which would help establish a more direct link between RG4‐mediated Nsp2 regulation and the antiviral activity of RG4‐targeting ligands.

In contrast, the weak responsiveness of M RG4 to the currently available ligands does not necessarily indicate a lack of biological relevance but may instead reflect its distinct structural features that limit efficient recognition by TMPyP4 or PDS [45, 46]. Considering the pivotal role of M protein in viral assembly [47, 48], further structural characterization of M RG4 and screening of more selective ligands will be valuable in future studies [12, 49].

It is important to note that the G4 ligands used in this study, such as TMPyP4, should primarily be regarded as tool compounds for mechanistic investigation rather than direct drug candidates, due to their limited sequence specificity, potential cytotoxicity, and unoptimized pharmacokinetic profiles [50]. In particular, their limited sequence specificity suggests that these compounds may not only target viral RG4 structures but also interact with host cellular DNA or RG4 structures. If such off‐target effects occur, they may perturb host G4‐regulated processes, including cellular gene expression programs, RNA stability, translational output, stress responses, and innate immune signaling. Since these host processes can influence the cellular environment required for viral replication or host antiviral defense, off‐target modulation of host G4s may indirectly affect PDCoV replication. Therefore, when interpreting the antiviral effects of G4 ligands, both their direct targeting of viral RG4s and potential host G4‐mediated off‐target effects should be considered. Building on these findings, future efforts should focus on developing highly selective and low‐toxicity small molecules that specifically target PDCoV RG4s. These improved ligands will help more accurately define the contribution of viral RG4 targeting to antiviral activity. Consistent with the experimental strategies used in many previous studies of viral RG4‐targeting compounds [18, 30–32], we first adopted a pretreatment‐plus‐continuous‐treatment regimen, in which cells were pretreated with G4 ligands before PDCoV infection and the compounds were maintained during the subsequent infection period, to evaluate the overall antiviral activity of these ligands. Nevertheless, as reported in the PEDV RG4 study [18], stage‐specific treatment assays can provide important information on whether G4 ligands affect viral attachment, internalization, replication, or later stages of the viral life cycle. Therefore, future postinfection‐only and time‐of‐addition experiments, in which ligands are added after viral infection has been initiated or at defined infection stages, will be needed to assess whether RG4 ligands can suppress ongoing PDCoV infection and to further define the stages of the PDCoV life cycle affected by these compounds. In parallel, reverse genetics approaches to engineer RG4‐Mut PDCoV strains will allow direct evaluation of the functional roles of RG4 structures in viral replication, protein expression, and pathogenesis in physiologically relevant systems such as porcine respiratory organoids and piglet models [19, 51]. These studies will elucidate the fundamental mechanisms of RG4‐mediated regulation in PDCoV biology, thereby providing a foundation for the rational design of RNA‐targeted antiviral strategies against emerging deltacoronaviruses (Figure 6F).

In summary, this study identified two cross‐strain highly conserved RG4‐forming sequences within the *Nsp2* and *M* genes of PDCoV and supported that both of them can fold into stable structures and exert translational repressive functions. Meanwhile, we found that the G4 ligands TMPyP4 and PDS exhibit antiviral activity and are capable of targeting the RG4 structure within the *Nsp2* mRNA, thereby suppressing the Nsp2‐EGFP expression. These findings deepen our understanding of RNA structure‐mediated regulatory mechanisms in coronaviruses and highlight viral RG4s as promising therapeutic targets. Given the high degree of conservation of these RG4 elements, RNA‐targeting strategies against PDCoV may represent a promising intervention approach that could reduce the risk of viral escape and lay the groundwork for the creation of effective antiviral therapies targeting this emerging swine pathogen.

## 5. Conclusions

This study identified two translationally functional RG4s in the PDCoV genome and demonstrated the antiviral activity of the G4 ligands TMPyP4 and PDS. Collectively, the findings suggest that conserved viral RG4s are potential RNA‐based antiviral targets and offer theoretical support for developing new therapeutic strategies against PDCoV.

## Funding

This work was supported by the National Natural Science Foundation of China (Grants 32372991 and 32000028) and the Fundamental Research Funds for the Central Universities (Grant KJJQ2025021).

## Ethics Statement

This study did not involve any animal or human subjects. All experiments were conducted using established cell lines. Therefore, ethical approval was not required.

## Conflicts of Interest

The authors declare no conflicts of interest.

## Supporting Information

Additional supporting information can be found online in the Supporting Information section.

## Supporting information


**Supporting Information** Table S1: Predicted PQS within the PDCoV genome identified using QGRS Mapper. Table S2: Sequences of oligonucleotides employed in this study. Figure S1: Thermal stability analysis of Nsp2‐ and M‐RG4s in the presence of G4 ligands. Figure S2: Posttranscriptional regulation of M expression by RG4. Figure S3: Flow cytometric analysis of the effects of two ligands on Nsp2‐EGFP expression levels.

## Data Availability

All data generated or analyzed during this study are accessible from the corresponding author upon reasonable request.
