# Supplementary material for: RNA G‐Quadruplexes in the Porcine Deltacoronavirus Genome: Structural Regulation and Therapeutic Targeting for Antiviral Strategies
Source: Transbound Emerg Dis. 2026 Jul 31;2026:5088147. doi: 10.1155/tbed/5088147 (PMC13426326; doi:10.1155/tbed/5088147)
Supplement: Supplementary file 1 — Supporting Information Table S1: Predicted PQS within the PDCoV genome identified using QGRS Mapper. Table S2: Sequences of oligonucleotides employed in this study. Figure S1: Thermal stability analysis of Nsp2‐ and M‐RG4s in the presence of G4 ligands. Figure S2: Posttranscriptional regulation of M expression by RG4. Figure S3: Flow cytometric analysis of the effects of two ligands on Nsp2‐EGFP expression levels. [file TBED-2026-5088147-s001.docx]

**Supplemental Information**

**RNA G-Quadruplexes in the Porcine Deltacoronavirus Genome:**

**Structural Regulation and Therapeutic Targeting for Antiviral**

**Strategies**

**Jiajing Guo^1,2^, Mengqi Yu^1,2^, Yue Sun^1,2^, Qiao Chen^1,2^, Fei Liu^1,2*^, and Yanke Shan^1,2*^**

*^1^College of Veterinary Medicine, Sanya Institute of Nanjing Agricultural University, Sanya 572000, Hainan, China*

*^2^Joint International Research Laboratory of Animal Health and Food Safety of Ministry of Education, Single Molecule Biochemistry & Biomedicine Laboratory (Sinmolab), Nanjing Agricultural University, Nanjing 210095, Jiangsu, China*

2022207001@stu.njau.edu.cn (Jiajing Guo); [ymq0411@stu.njau.edu.cn (Mengqi Yu);](mailto:1669256684@qq.com(M.Yu.);) [2024107015@stu.njau.edu.cn (Yue Sun);](mailto:2024107015@stu.njau.edu.cn;) [chenqqqqq2024@163.com](mailto:chenqqqqq2024@163.com) (Qiao Chen)

**Correspondence should be addressed to Fei Liu; feiliu24@njau.edu.cn, and Yanke Shan (Submitting author); shanyk26@njau.edu.cn**

**Table S1 Predicted PQS within the PDCoV genome identified using QGRS Mapper.**

| **Position** | **Length** | **QGRS** | **G-Score** |
| --- | --- | --- | --- |
| **788** | **28** | GGTGTGTGAGGCTCGCTCGGCTGAATGG | **20** |
| **939** | **28** | GGCCGATTGGTTAAAATTGAGGACAAGG | **15** |
| **1673** | **20** | GGTAGGTAGGCTCTATAAGG | **15** |
| **2600** | **27** | GGCTATGGAAGGTGAAGATGATAGTGG | **10** |
| **2807** | **17** | GGTTGGCAATGTTGGGG | **14** |
| **2856** | **25** | GGGGACCCAGTACCGGATCCTGAGG | **11** |
| **7967** | **30** | GGCTTTGTCCTCTTTTCGTGAAGGAGGCGG | **2** |
| **9500** | **27** | GGCTGCTCTTAGCTGGTGGCTAGCTGG | **10** |
| **10235** | **20** | GGCGGAATGGGAGCGTGAGG | **15** |
| **11244** | **29** | GGTGTTGATGGACGGTGCCCCTACAAAGG | **11** |
| **13154** | **27** | GGAACAACCAAATTCTATGGTGGTTGG | **6** |
| **15887** | **25** | GGTATCCTTGTGGTGTTTAGGCAGG | **14** |
| **16432** | **29** | GGACATGCGCCAGGCGCGTCCTTGGAAGG | **13** |
| **16604** | **26** | GGCAGGCGGGCTTGTTTCACTAATGG | **9** |
| **16711** | **25** | GGCTACGTGGGGATTCTCGGGACGG | **17** |
| **16957** | **30** | GGTGGTTCACGATATTGGTAACCCAAAAGG | **11** |
| **18924** | **28** | GGATAGTTAAGGAGAGGCTCGCCCTCGG | **15** |
| **19096** | **27** | GGGGCTTGCTAAGGAAAACATTAATGG | **10** |
| **19985** | **21** | GGTTGTATCGGGGTCAATTGG | **14** |
| **21514** | **30** | GGCTCTCTTACTGGAGCTATGGTATTTGGG | **17** |
| **22684** | **23** | GGTTGTTGTGGTGGTTGTTTTGG | **15** |
| **23085** | **27** | GGCGCTTGGCGTCATCCTCCAAGGAGG | **9** |
| **23365** | **27** | GGGATTATGGATCCAATGGGTACATGG | **20** |
| **24431** | **26** | GGAAGACCTCAGGAGCGTGGAAGTGG | **16** |
| **24598** | **27** | GGTATTTGGCAACCGGTCTCGTACTGG | **17** |
| **25341** | **29** | GGTTACAATTGTGGTGGGGACAAATTAGG | **14** |
| **25370** | **30** | GGAAATTAAATTGGCTTATAGGGGGGATGG | **17** |

*The red font indicates the overlapping sequence between QGRS Mapper and G4Hunter.

**Table S2 Sequences of oligonucleotides employed in this study.**

| **Name** | **Squences** |
| --- | --- |
| *NSP*2-F (qPCR) | GTCCACACTTGCTGCCTTTG |
| *NSP*2-R (qPCR) | GATAGTGGCGCGAGGGTAAA |
| *M*-F (qPCR) | ATGTCTGACGCAGAAGAGTGG |
| *M*-R (qPCR) | GGTCACCACTAGGGTGAAGG |
| Nsp2 PQS WT | GGUGUGUGAGGCUCGCUCGGCUGAAUGG |
| Nsp2 PQS Mut | GAUGUGUGAGACUCGCUCGACUGAAUAG |
| M PQS WT | GGGAUUAUGGAUCCAAUGGGUACAUGG |
| M PQS Mut | GAGAUUAUGAAUCCAAUGAGUACAUAG |


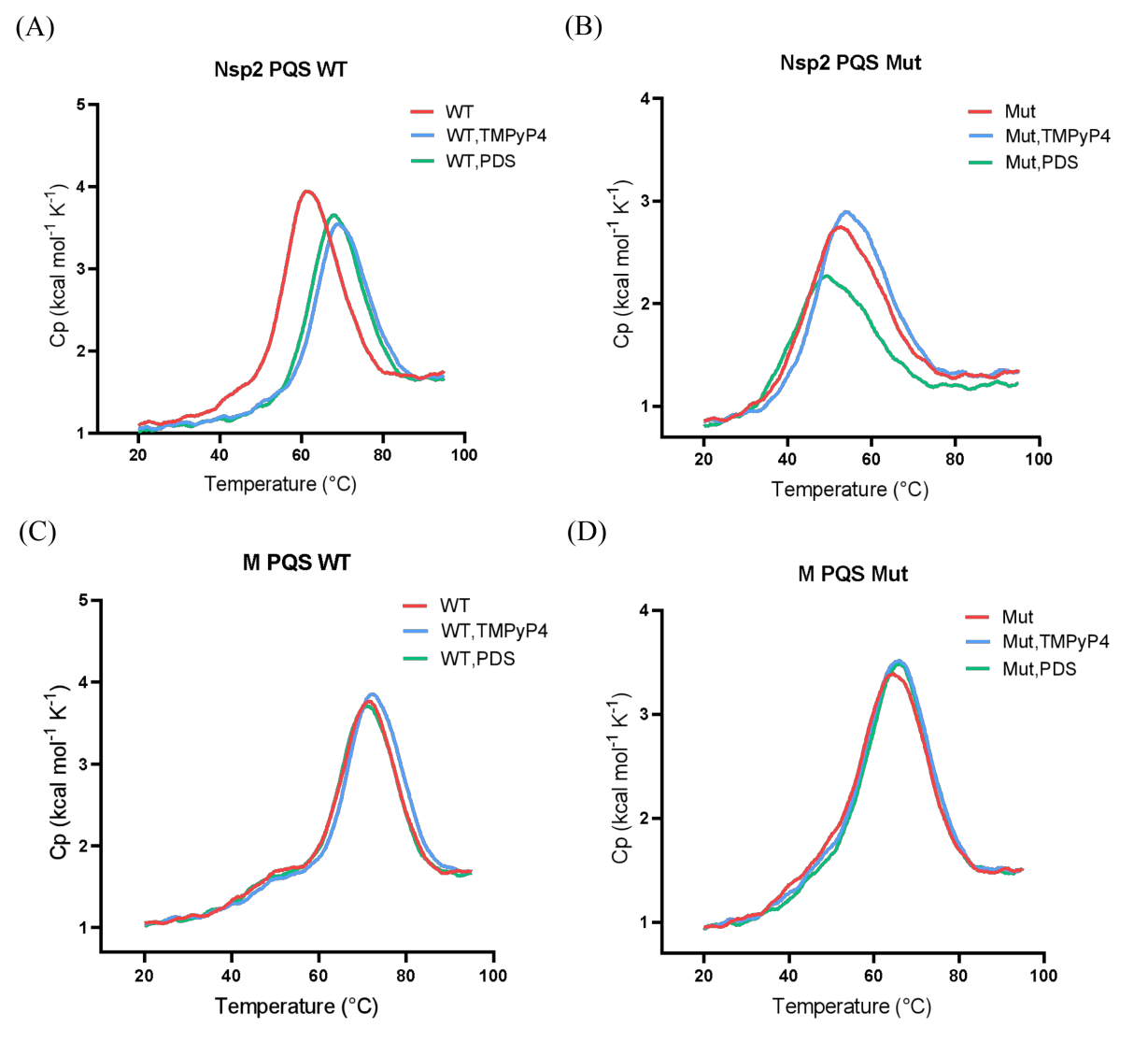


**FIGURE S1. Thermal stability analysis of Nsp2- and M- RG4s in the presence of G4 ligands.** DSC thermograms of Nsp2-PQS-WT (A), Nsp2-PQS-Mut (B), M-PQS-WT (C), and M-PQS-Mut (D) in the absence or presence of TMPyP4 or PDS. RNA oligonucleotides were analyzed at a final concentration of 150 μM, and DSC scans were performed at a heating rate of 1°C min⁻¹.

**
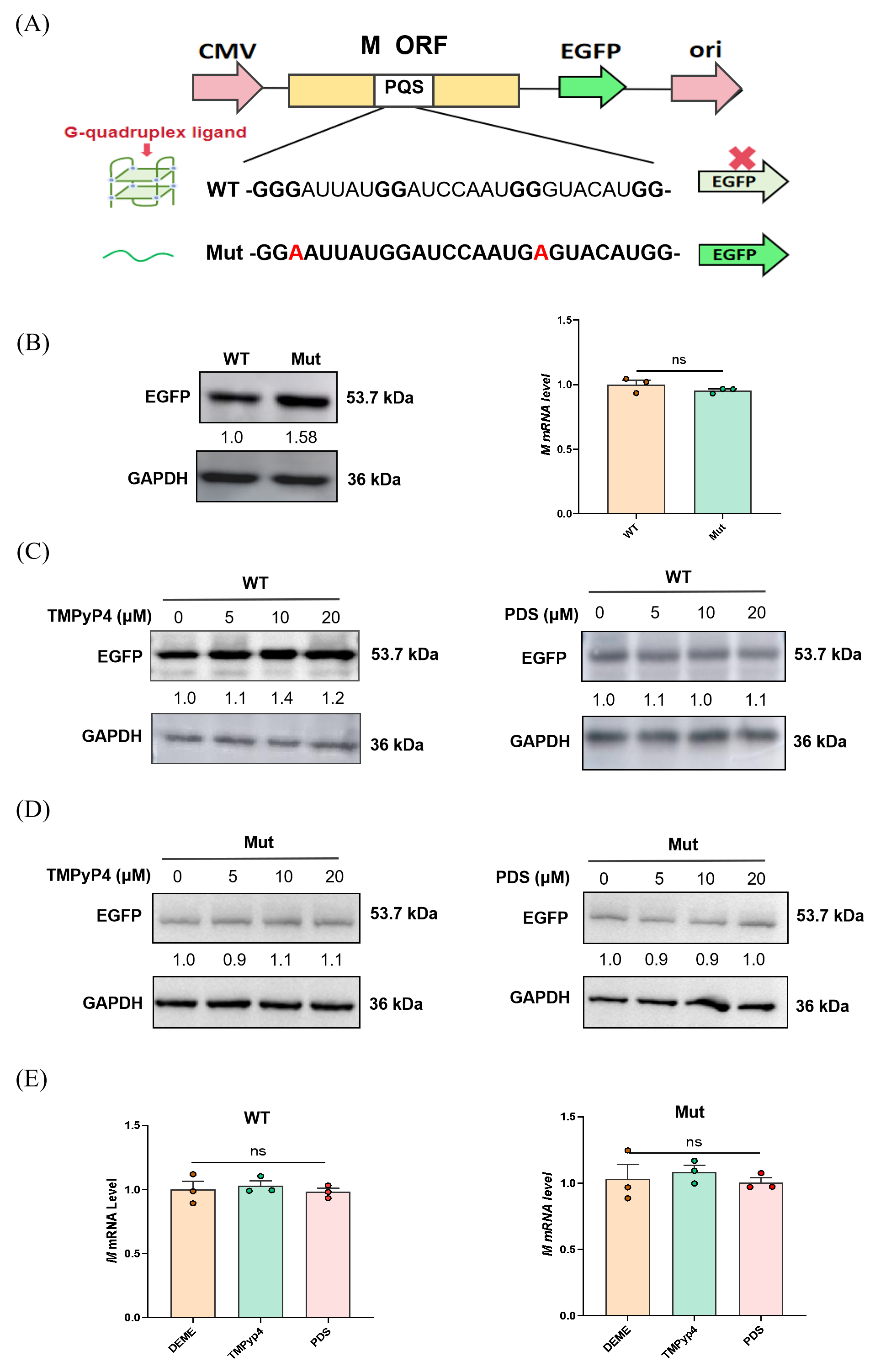
**

**FIGURE S2 Post-transcriptional regulation of M expression by RG4.** (A) Schematic of *M* full length ORF or RG4 mutant constructs cloned into the MCS of pEGFP-N1. Bold letters​indicate RG4-forming sequences;​red letters denote mutations.​(B) M-EGFP protein (left panel) and mRNA (right panel) levels in cells transfected with pEGFP-M-WT or Mut plasmids.​(C) pEGFP-M-WT or Mut (D) protein levels in cells treated with increasing concentrations of TMPyP4 (left) or PDS (right) for 24 h.​(E)​qPCR quantification of pEGFP-M-WT (left) or Mut (right) mRNA levels in cells treated with G4 ligands (20 μM) for 24 h.

**
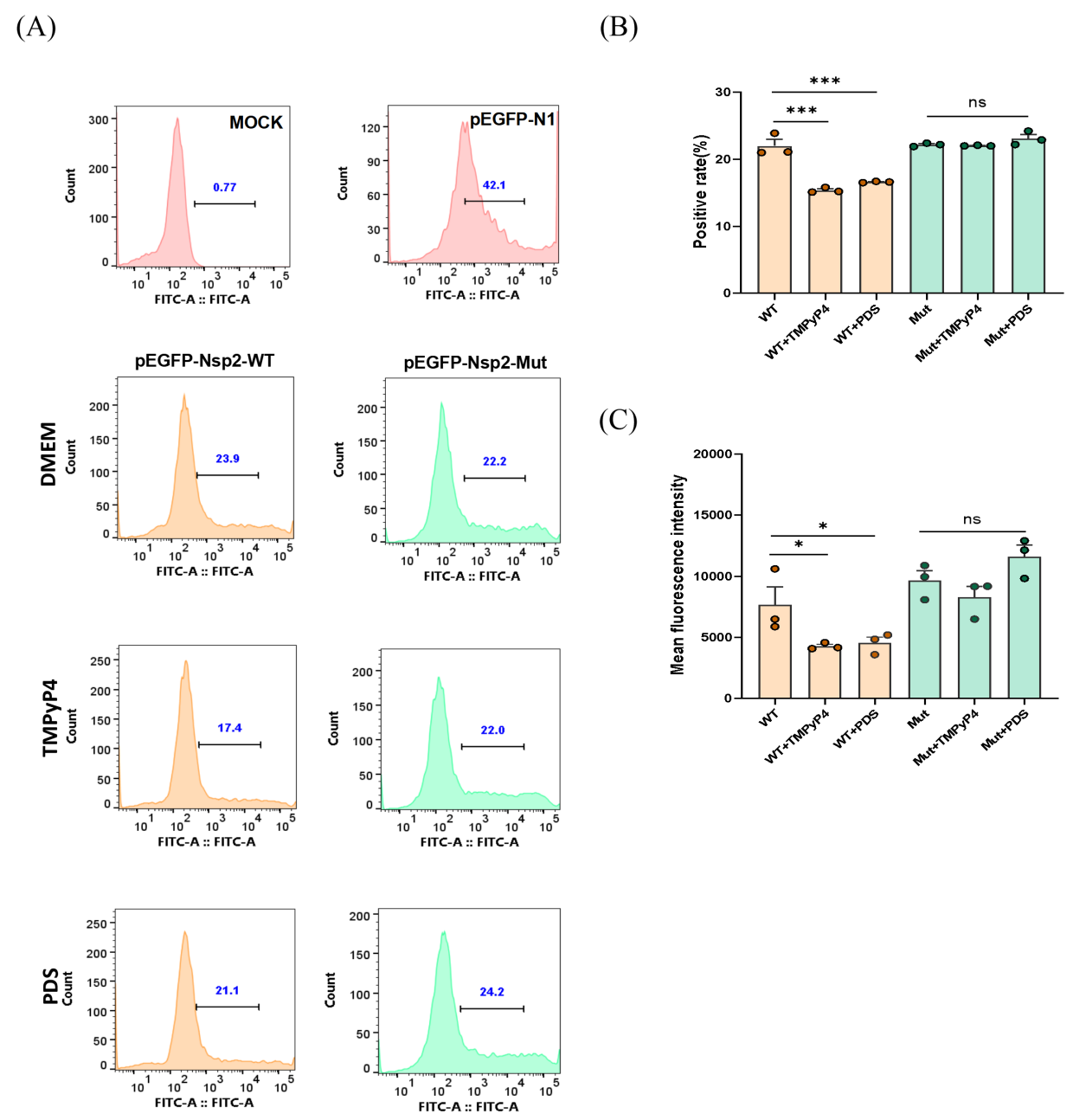
**

**FIGURE S3 Flow cytometric analysis of the effects of two ligands on Nsp2-EGFP expression levels.** (A) Cells transfected with either pEGFP-Nsp2-WT/Mut were incubated with TMPyP4, PDS, or DMEM for 24 h. (B) The percentage of positive cells and (C) MFI were measured using flow cytometry.
